# The task before psychiatry today

**DOI:** 10.4103/0019-5545.31521

**Published:** 2007

**Authors:** Ajai R. Singh

**Affiliations:** Psychiatrist. Earlier, Senior Research Fellow, WHO Collaborating Center in Psychopharmacology in India

## INTRODUCTION

While each one of us has his own special concerns and would like to give his own take on what he considers the task before psychiatry today, I want to highlight some critical areas for your consideration which are likely to be common concerns for us all.

I divide them into five tasks to be performed, four of them of concern to all psychiatrists everywhere and a fifth of special concern to Indian psychiatrists and psychiatry.

## THE FIRST TASK: SPEAK TO A WIDER AUDIENCE ABOUT POSITIVE CONTRIBUTIONS OF PSYCHIATRY

Just do a Google/Yahoo/MSN search on ‘Psychiatry’, ‘Positive Aspects of Psychiatry’, ‘Anti-Psychiatry’. You will get a lot of matter to browse. Please do. It will be an eye-opener.

While you will get to read matter on positive contributions of the branch, quite a lot of it would be about negative aspects of psychiatry, how it is useless and dangerous, how it harms and is an infringement on human rights, free will etc, how barbaric is ECT treatment, how dangerous are its medications etc. It goes on and on and on. And while you and I sit in our consulting rooms and our OPDs and our seminars and conferences and committees and task forces, debating this and that issue and ostensibly working for patient welfare and therapeutic advancement in psychiatry, a major part of the world around remains unimpressed and keeps ranting about the ill-effects of our branch.

### Psychiatry and its critics

There is something about psychiatry that attracts the most vehement protests. No other branch of medicine sees such vilification heaped on it.

And yet, those who are in the system know they are doing the best thing possible for their patients/clients. And it is the one system that is most open in discussing what needs to be improved about it. While many other systems of medicine would dismiss most protests with a shrug, psychiatry is one branch that considers ethical, conceptual and foundational issues, sometimes almost to the point of becoming paralysed for action due to this.

Every psychiatrist knows the benefits of ECT in selected patients. Every psychiatrist knows how psychopharmacology has revolutionised patient care. The grateful patients who have been saved from suicide, who get rid of their delusions/anxieties/phobias/depressions to lead a productive life, whose inter-personal and intra-personal problems have been resolved with psychotherapy - all these are so very well known in psychiatric practice. And yet the vilification of the branch continues.

Of course not all of anti-psychiatry has been useless and only vitriol. One can understand the contributions it has made to mental health consumerism. And it has added enlighteningly provocative critiques to ‘establishment’ psychiatry in the form of thinkers like Michel Foucault in France, R. D. Laing in Great Britain, Thomas Szasz in the United States and Franco Basaglia in Italy.[1] It essentially champions personal reality and freedom and that is a laudable approach. But it also opposes any attempt at a definition of normalcy that, according to it, the establishment of psychiatry tries to ‘impose’ on nonconformists and society at large. Here it transgresses its legitimate domain, for a definition of normalcy is essential, although difficult, as is a definition of the abnormal. And every non-conformism is not legitimate. That which confronts social prejudices and stereotypy is; but that which tries to legitimize people's disease and resultant distress, suffering and anguish in the name of non-conformity, isn't what we label as psychiatric disorders falls in the latter category. Moreover, the inability to precisely define a certain phenomena, as for example the difference between normal and abnormal, does not mean it does not exist. It only requires a more clear-cut delineation, which researchers need to work over more diligently. Studies on normality need greater emphasis than is their lot at present.

While every attempt must be made to engage energies in such definition, it is more necessary to move on and outline the processes and manifestations of abnormality in the different forms that it takes and find appropriate methods of treatment and care. In other words, major energies must be concentrated on the aetio-pathology, diagnostic finesse and treatment of the myriad psychiatric disorders. And define and treat them with greater and greater precision.

### Patients who get well

The whole problem also is patients who get well do not talk. They go on with leading their lives and often want to hide their psychiatric history for fear of stigma.

It's a rare instance that a man would speak as eloquently about his psychosis and how he got rid of it, as he would about his recent bypass, or appendectomy, or whatever.

It's not that treatment failures do not occur in other branches of medicine. But they are accepted as part of the process. No one wants them. But no one dies a thousand deaths over them. However, in psychiatry, its opponents trumpet every treatment failure so loud as to scare so many more who would greatly benefit by it.

Mercifully, the anger and vituperative outbursts of some of the earlier critics of psychiatry seem to show signs of abating. They have, in a way, found ways to sublimate their anger and aggression. It is encouraging to note that most anti-psychiatry today is not as keen to focus on dismantling organized psychiatry as on seeking to promote radical consumerist reform.[1] Good for them and good for us too.

However, a substantial section of the disgruntled, which possibly also includes those incompletely treated, continue to relentlessly pour acid comments on the branch.

### The easiest option and what do we do

What do we do? The easiest option is to do nothing about it. And that's what most of us probably do. Or go on doing one's bit to the best of one's ability. And think of the grateful faces of those helped. And wait for saner counsel to prevail in the less charitably disposed.

That's a good ploy to keep one's equilibrium in the face of acerbic comments. But it's hardly likely to counter their thrust. And it's hardly likely to help motivate those who can be helped by psychiatric therapy to seek it. In fact, negative comments about the branch, or its practitioners, have the uncanny ability to dissuade the needy from seeking help, even if they continue to suffer because of such a refusal.

The remedy for this is not just remaining quiet and doing one's work. The remedy is asking oneself this simple question: if every psychiatrist knows the benefits his patients get by his treatment, what does he do to let the world around know that his methods work?

I would, therefore, urge you to give up on your slumbers and make some effort to list the positive contributions of our branch in general and your own in particular, to make life worthwhile for the psychiatric patient.

It's not just enough to be convinced yourself about the worth of your branch. That is important, but not enough. It's also important the tirade against psychiatry is fittingly countered. By clear cut therapeutic evidence, by patient data, by statistical details, by replicated studies.

### Speak up, patients and clinicians

Maybe it is also time for those who have been helped by psychiatry to speak up.

Patients who get well are grateful, but often just remain anonymous. Fear of stigma makes them become just part of a statistic, in a clinic, or hospital, or research paper. They are apprehensive about speaking out aloud about the benefits of treatment. The disgruntled elements have no such compunctions. So they jolly well shout from roof-tops. And the e-world is inundated with their vitriol. It's time you and I did something to mend matters.

Pick up your pen. Write about how psychiatry helps. If possible, put up a web site, alone or in a group, where authentic information and guidance about psychiatry is available and especially highlight what are its positive contributions. Not propagandist, not evangelical. Just the facts.

Let patients and patient groups, who are benefited, speak. Let those who are not afraid to come out of the closet and have the necessary ability to communicate get a platform to talk how psychiatry has helped them. Let others who are helped but are diffident about coming out in the open find other avenues (anonymous) to voice their opinion.

A regular column in the *Indian Journal of Psychiatry a* nd other psychiatry journals around the globe, called ‘Patients Speak’ or something similar, wherein those who have been helped get a chance to voice their stories, would be a step in the right direction. Occasionally, they may also voice their concerns, difficulties and criticisms; but only *occasionally* and never as a means to emasculate the branch, or deride fellow practitioners.

This is the first task before psychiatry and psychiatrists, today.

## THE SECOND TASK: SHRUG AMBIVALENCE AND DISAGREEMENT; SEARCH COMMONALITIES IN PSYCHIATRIC PHENOMENA

There is a lot of ambivalence about the contributions of psychiatry in the minds of psychiatrists themselves. There is more disparity in thought and approach about almost every psychiatric disorder and therapy than there are drops in the Indian Ocean.

Psychiatrists seem to thrive on disagreement. They stress individuality almost to the point of denying any commonality and scientific categorization. While it is true that each patient is unique and requires individual handling, he is also part of the human race, which has many things in common. Reconciling the nomothetic-idiographic dichotomy, the IGDA mentions:

*The diagnosis itself should combine a nomothetic or standardised diagnostic formulation (e.g. ICD-10, DSM IV) with an idiographic (personalised) diagnostic formulation, reflecting the uniqueness of the patient's personal experience*.'[2,3]

However, an idiographic orientation which stresses individuality cannot and should not, preclude the nomothetic or norm laying thrust that is the crux of scientific progress. The major contribution of science has been to recognize such commonalities so they can be researched, categorized and used for human welfare.

What is the difference between a lay intelligent observer and a scientist? A lay intelligent observer would try to find out the individual variations and peculiarities of abnormal behaviour as it manifests in different individuals and different cultures. A scientist will try to decipher the commonalities in the abnormal behaviour across cultures and peoples so he can find stable symptom clusters that can be labeled as diseases/syndromes etc. Which then help him decide a plan of therapy and delineate the course and outcome of the said disease/syndrome.

It is a mistake to stress individuality so much that commonalities are obliterated. For that is counter-scientific. Well meaning psychiatrists will have to be especially careful they do not carry their honestly intentioned emphasis on individuality to ridiculous limits. This is what makes some of them come dangerously close to being unscientific themselves and makes a few, if not most of them, consort with willing accomplices from the anti-psychiatry group. In fact, most of those in the other group are basically anti-science, as applied to psychiatry. This means they somehow consider the scientific method as unsuited and inadequate for the psychiatric approach. That is their fundamental peeve. It's a conceptually flawed position, precisely because psychiatry is a branch of care where patients, therapy and sickness is involved. And while holistic understanding is necessary to study intimate nuances of psychological/psychopathological processes and while individual manifestations and individual approach are laudable goals in treatment and approach, we cannot forget that major therapeutic advances result from being able to delineate commonalities and stable symptom clusters that are amenable to study and intervention.

Let us appreciate the fact that although stress on the individual's needs has helped psychiatry at times become more humane, it has hurt the task enormously by making some very bright minds question the very scientific basis of psychiatry. There can be no doubt on the issue that while the purpose and approach of psychiatry, as of all medicine, has to be humane and caring, the therapeutic advancements and aetiologic understandings are going to result only from a scientific methodology.

The art is in the caring approach. The touch, the tone, the outlook. The total attitude of the care-giver and the way that care is administered. Here all medicine, psychiatry included, is an art. But it is a science in the methods used. Both in therapy and diagnosis. Let both these complement each other, but not blur boundaries. The art cannot become the approach itself. Just caring is not enough, if you have not mastered the methods of care, which only science can supply to medicine in general and psychiatry in particular (since we are concerned with it here). This message must go clear into our minds.

## THE THIRD TASK: PSYCHIATRISTS MUST CONTINUE TO DO PSYCHOTHERAPY

The psychotherapeutic approach holds tremendous potential as a therapeutic tool in psychiatry. But it is a myriad bunch of therapies, sometimes so diverse and so disparate that it arouses doubt whether it is scientific after all. At times it seems to be a free for all. This state of affairs needs to be speedily remedied.

Psychotherapy must be clearly defined, its parameters and methods firmly delineated, its proof of effectiveness convincingly demonstrated. We know it suffers from a major resource constraint at present. That's because an important funding source, like the pharmaceutical or the medical device industry, is hardly likely to further genuine research in psychotherapy. And we know that's simply because it would be counterproductive to their very existence, based as it is on furthering the biological approach. But that is all the more reason some sincere researchers and altruistic sponsors, as also professional societies and governments themselves, become catalysts in this direction. It is important to search out such and it is important that professional societies, flush as they are with funds they often don't know what good to do with, take up such worthy causes. For example, the Indian Psychiatric Society has handsome funds lying in its kitty which it keeps multiplying in fixed deposits. Nothing wrong with that. But it is high time part of such funds were mobilized to further research fellowships in neglected areas of research, like psychotherapy and social psychiatry. Some salvaging of pride and retribution of guilt for the massive pharma funding that makes for Association funds. And what is applicable here is equally applicable for other professional societies with funds. What use money if not put to proper use?

### What proponents of psychotherapy must do

Equally importantly, proponents of psychotherapy will have to give up on their cynical disregard for the positive contributions of the biological approach. They will also have to forsake their archaic methods to prove effectiveness and stop being impressionistic and subjective, or sit in their cocoons idolizing their approach. They will have to provide enough irrefutable evidence that their methods work. And enough replicable studies that prove it across geographical areas.

The time to sing platitudes for the psychotherapeutic approach, or lament its neglect in circles of contemporary influence, is past. Only solid evidential basis will sustain it, or any other branch, in the future. As Singh and Singh mention:

*Ultimately, the psychotherapeutic approach itself will benefit by shedding its smug somnolence, become more evidence and experiment based, offer verifiable population statistics to back up its contentions and compete with biological approaches with greater methodological rigour.*[4]

Anyone who is only a psychopharmacologist is only half a psychiatrist. But anyone who is only a psychotherapist is equally half a psychiatrist. The total picture is painted only when both approaches are judiciously merged in the training and application of the individual psychiatrist and his branch itself.

Let's not get carried away by the sloganeering on either side. Let the committed proponent of either branch not find any virtue in such of his commitment as makes him oblivious to the strengths of his opponent and shortcomings of his preferred approach. In fact, his commitment should, if at all, make him acutely aware of the shortcomings of his preferred approach and make him equally acutely aware of the merits of his opponents'.[4]

### Handing over psychotherapy to clinical psychologists

Also, it will not do to hand over psychotherapy to clinical psychologists and others. Psychiatrists may enjoy priding themselves in becoming doctors in the sense they are commonly understood, when they mainly prescribe drugs and carry out ‘medical’ procedures and interventions. Psychiatrists may feel justifiably proud in prescribing drugs and giving ECTs. They would not want to hand over these responsibilities to others, would they, for they consider them their legitimate areas of expertise. Then why do they find it justified handing over doing psychotherapy to others? If lack of time is the justification, will they say the same when it comes to drug prescribing and ECTs? Will they hand over ECT treatment to trained technicians, or drug treatment to trained pharmacologists just because they don't have the time? Let's stop kidding ourselves.

The fact of the matter is psychotherapy is time consuming, taxing on the mental resources of the therapist and doesn't offer clear-cut tangible rewards. Moreover, it's not fashionable to be considered a psychotherapist; while it is an ego-booster to say that one has a couple or more of trained clinical psychologists in one's team to handle psycho-social issues. That may do good to the psychiatrist's self-esteem, but it does hardly any good to his credibility and standing as one.

In sum, then, it makes eminent sense for psychiatrists to continue to do psychotherapy. And it makes equally good sense for them to encourage/demand for well-designed studies that compare and contrast the different forms of psychotherapies and offer clear-cut guidelines of effectiveness and approach.

## THE FOURTH TASK: WELCOME BIOLOGICAL BREAK-THROUGHS, SUPPLY PSYCHOSOCIAL INSIGHTS

There is a huge mass of research outpouring in biological psychiatry. And not without reason. The quest to find biochemical and neurophysiological underpinnings of mental phenomena in health and disease is a legitimate exercise and likely to yield fruitful results for aetiopathologial and therapeutic advance. The biological approach in psychiatry holds great promise to someday unravel the intricacies and mysteries of the brain in health and disease, a promise no other approach holds as much.

### Breakthroughs from biology, insights from the psychosocial and philosophical

While the experimental breakthroughs, both in aetiology and therapeutics, will come mainly from biology, the insights and leads can hopefully come from many other fields, especially the psychosocial and philosophical. It is in some such synergy that these two supposedly antagonistic branches must engage themselves, to complement and nurture rather than confront and dismember. Nobel Laureate Kandel's dream of the integration of cognitive neurosciences with scientific psychoanalysis[5] and the wish that psychoanalysis reenergize itself by developing a closer relationship with biology in general and cognitive neuroscience in particular is worth a close look in this connection.[6]

Integration of approaches is essential for a complete psychiatrist. He has no option, really. The biological and the psychological are not exclusive but complementary approaches.[4] There can be no changes in behaviour that are not reflected in the nervous system and no persistent changes in the nervous system that are not reflected in structural changes on some level of resolution.[5] An insightful comment, which sums up what is the essence of the integrative approach for the psychiatrist, is the one by Gabbard:

*Just as the physicist must simultaneously think in terms of particles and waves, the psychiatrist must speak of motives, wishes and meanings in the same breath as genes, neurochemistry and pharmacokinetics.*[7]

### Integration and reductionism: Both valid

However, although integration of approaches is necessary, the reductionist approach of biology is eminently suited to aetiologic understandings as well as therapeutic breakthroughs. Often, too many approaches result in a multitude of viewpoints that obscure and mystify rather than simplify and clarify phenomena. The aetiology and definitive therapy of major conditions in psychiatry would have been known earlier if we were not bombarded by a plethora of conceptual formulations that, in the name of justifying how complex and mysterious the mind is, only obfuscate issues and make the terrain so much more difficult to tread. If the scientific approach is robustly furthered and the reductionist/replicable approach firmly adhered to, significant insights into aetiology and therapy of major psychiatric conditions will yield themselves to the keen researchers' probings.

Hence, although integration is necessary as an attitude, reductionism is necessary as an approach. Both must co-exist in the individual psychiatrist, as much as the branch itself.

## THE FIFTH TASK: PROMOTE GENUINE RESEARCH ALONE AND WORK TOWARDS

A lot of research in psychiatry is substandard, especially in India. While we may probe the reasons why it is so, the major cause is a mind-set that does not allow for a conviction that anything trend setting can result from here. This scenario will hopefully change in the next few years as the Indian has now developed a new-found confidence in his own abilities and, over the next decade or two, will get rid of his awe and subservience to the western mind. The changed economic and socio-political climate, with the manner in which Indian enterprise is making its mark on the world scene in most walks of life, as also the fact that Indians are no longer treated with outright contempt or disguised condescension abroad, something that was their lot till very recently – all these have made for a firm current of positive self-esteem in the Indian of today. It will not take long for this current to become a torrent in the next decade. And it should not take long for this heightened self-esteem to percolate to the scientific and research fields too. Indian medicine and psychiatry should be its major beneficiaries.

### The task for Psychiatry departments and research units in India

The task before major departments and research units in psychiatry all over the country is to respond to this change with vigour and conviction. The need is to stop engaging in poor quality research. The need is to stop waiting for the next important discovery to come from the West. The need is to believe in oneself and become the next Center from where important breakthroughs can result.

For that it is necessary for heads of units to tighten their belts. To stop promoting poor quality research. And researchers. To stop encouraging sycophants and ladder climbers. To pick up and hone genuine research talent from amongst their faculty and students. Let us not rue the fact that promising talent seeks to run to the West for brighter pastures there. This is inevitable for some more time. But if we offer them comparable research facilities and a clean work environment that encourages and hones talent - and with the changed economic and political scenario that has unshackled the economy and promotes individual growth - it is very much possible more and more genuinely talented researchers and clinicians would want to stay. The important thing is for heads of units and policy makers and decision takers not to get cynical and give up on promoting excellence in their respective work places because some promising talent drains away.

If we are persistent enough and offer quality setups, the time when talent will come back seeking work here is also not far away. And the brain drain will get stemmed as well. But all this will not happen by itself. There is no Santa who will offer it as a gift. It will only result by developing consistent quality environs in the departments and having Heads of Units who recognize, hone and nurture talent. And who never give in to pessimism and cynicism.

### An Indian Nobel laureate in Psychiatry by 2020

I am no soothsayer, but there is no doubt in my mind that there will be a Nobel Laureate in medicine/physiology of medicine from India in the next fifteen years. 2020 or thereabouts. We have to decide if this Nobel Laureate will be from the field of psychiatry.

It is not that difficult a task. But it will only happen if a number of psychiatric departments all over the country are engaged in quality research for the next few decades.

Which department will produce the Nobel Laureate we may leave to destiny. But it will only result when most departments believe it is within the realm of possibility and put in earnest efforts to become the chosen one.

When a stable wants to produce champion thoroughbreds, it does not bank on a single horse. It identifies a number of potential champions, hones and nurtures them, with care and caution. And waits for one or more from the brightest to win the race for them.

Similarly, a number of psychiatric research departments and research institutes will have to engage in top quality research for a couple of decades for some champion from amongst the many thoroughbreds so produced to win the race for them.

If you are one who heads such a department, you know the task that is cut out for you. If you are one who has such a winner in sight, or as a member of your department, you know what you have to do. And if you are one who believes you can be one such potential winner, you know the terrain you must traverse.

Just stop doing, and promoting, poor quality research work.Just stop the temptation to earn a quick buck by doing the next drug trial. Just stop being satisfied with the money, power and prestige that comes by wheeling-dealing, groupism and politicking. And stop wasting energies in Association politics. Association work is fine and necessary and laudable; but that too within limits, if doing quality research is your objective. Association politics, however, is to be firmly avoided, simply because it will drain you, emotionally and intellectually; and render you incapable of visualizing that anything higher than an official position is even possible. You have greater goals to achieve and nobler tasks to perform. And they are within your grasp if you just reach out hard enough and persistently enough.

## CONCLUSION

We entered the branch of psychiatry with a hope and desire to make a difference in the lives of our patients and work to fathom the mysteries of the mind. As Nancy Andreasen asks: why did we become psychiatrists and not cardiologists, radiologists, pathologists and surgeons? It's because we were interested in understanding what makes human beings tick;[8] in health and disease:

*Every person whom we encounter is a new adventure, a new voyage of discovery, a new life story, a new person… We are privileged to explore the most private and personal aspects of people's lives and to try to help them become healthier.*[8]

Someone down the line, however, most of us got bogged down with the nitty-gritty of living and ‘achieving’ and laurels and power and prestige. The glorious vision that made us take up the branch got blurred under the foliage of false leads and superfluous achievements. And a crippling cynicism that hurts any worthwhile foray into genuine research, or appreciating someone else who is so engaged.

Let's clear the dust from the picture. The radiance is still there. The magic can be recaptured, to guide and illumine the path to brilliance. Infinite vistas of opportunity wait in the wings to unfold and offer opportunities for unraveling the mysteries of the mind to the earnest seeker. Provided he is ready to seek the valuable. Provided he stops holding on to the artificial and the superfluous. Provided he believes he deserves the very best and will not compromise on anything lesser, come what may.

It's a big game, my friends. Learn to play it big. The smaller fishes and loaves, of office and career and position and lucre, will pale into insignificance when you realize what larger issues beckon you to put your shoulder to the wheel.

Are you ready?

## SUMMING UP

The following are the five tasks before psychiatrists and psychiatry today:

- Speak to a wider audience about positive contributions of psychiatry
- Shrug ambivalence and disagreement; search commonalities in psychiatric phenomena
- Psychiatrists must continue to do psychotherapy
- Welcome biological breakthroughs, supply psychosocial insights
- Promote genuine research alone and work towards an Indian Nobel Laureate in psychiatry By 2020
